# Divergent artificial selection for female reproductive investment has a sexually concordant effect on male reproductive success

**DOI:** 10.1002/evl3.21

**Published:** 2017-08-23

**Authors:** Joel L. Pick, Pascale Hutter, Barbara Tschirren

**Affiliations:** ^1^ Department of Evolutionary Biology and Environmental Studies University of Zurich Winterthurerstrasse 190 8057 Zurich Switzerland; ^2^ Department of Animal and Plant Sciences University of Sheffield Western Bank Sheffield S10 2TN United Kingdom; ^3^ School of Biological, Earth and Environmental Sciences University of New South Wales Randwick, NSW 2052 Sydney Australia; ^4^ Centre for Ecology and Conservation University of Exeter Penryn TR10 9FE United Kingdom

**Keywords:** Egg size, fertility, fitness, maternal investment, paternity, sex‐specific selection, sexually concordant selection, testis asymmetry, testis size

## Abstract

Depending on the genetic architecture of male and female fitness, sex‐specific selection can have negative, positive, or neutral consequences for the opposite sex. Theory predicts that conflict between male and female function may drive the breakdown of intrasexual genetic correlations, allowing sexual dimorphism in sexually antagonistic traits. Reproductive traits are the epitome of this, showing highly differentiated proximate functions between the sexes. Here we use divergent artificial selection lines for female reproductive investment to test how female‐specific selection on a sex‐limited trait affects male reproductive success in a precocial bird, the Japanese quail (*Coturnix japonica*). We demonstrate that selection for increased egg investment in females positively affects male reproductive success both in competitive and non‐competitive mating situations. This increased reproductive success was linked to a relatively larger left testis in males originating from lines selected for high female reproductive investment. Given that female quail have functional gonads only on their left side, this correlated response indicates that selection has acted on the shared developmental basis of male and female gonads. Our study thereby provides evidence for a positive genetic correlation between key reproductive traits in males and females despite a high degree of sexual dimorphism, and suggests that, in this system, selection on reproductive function is sexually concordant.

## Introduction

In sexually reproducing organisms, males and females are adapted for their respective roles in reproduction. Selection therefore often acts in a sex‐specific manner, to the adaptive benefit of that particular sex. However, given that the two sexes share the majority of their genome, such sex‐specific selection is likely to impact the phenotype of the other sex, and, most likely, therefore their fitness (Lande 1979; Poissant et al. 2010). The consequence of such (positive) intersexual genetic correlations, arising from shared genetic variation between the sexes, depends on whether the fitness optima for the two sexes lie in the same direction relative to their current phenotypes. If so (i.e., selection is acting sexually concordantly) such intrasexual genetic correlations will result in an amplification of the selection response in both sexes and the purging of deleterious alleles from the population (Whitlock and Agrawal 2009). If, however, fitness optima differ between the sexes, (i.e., selection is acting sexually antagonistically) intersexual genetic correlations will result in an intralocus sexual conflict and contribute to the maintenance of variation in male and female traits (Lande 1979; Chippindale et al. 2001; Fedorka and Mousseau 2004; Foerster et al. 2007).

On an evolutionary timescale, intralocus sexual conflict is predicted to drive the two sexes to evolve a separate genetic basis (sex‐specific genetic variation; Lande 1979; Poissant et al. 2010) or sex‐specific patterns of gene expression for shared traits (Bonduriansky and Chenoweth 2009). By removing intersexual genetic correlations, this allows both sexes to reach their sex‐specific fitness optimum and will result in strong sexual dimorphism in the trait in question. In this light, sexual dimorphism, and in its most extreme manifestation, sex‐limited trait expression is often seen as an indication of past sexual conflict (Cox and Calsbeek 2009). This hypothesis is supported by a positive correlation between sexually antagonistic selection (i.e., divergent fitness optima between the sexes) and sexual dimorphism (Cox and Calsbeek 2009), and a negative correlation between sexual dimorphism and intersexual genetic correlation (Bonduriansky and Rowe 2005; Coyne et al. 2008; Poissant et al. 2010) across traits and studies. Furthermore, in species where sexual dimorphism increases through ontogeny, sex‐specific gene expression increases, whereas intersexual genetic correlations diminish (Cox et al. 2017). Therefore, sex‐specific selection on highly dimorphic or even sex‐limited traits is predicted to have little impact on the other sex (but see Harano et al. 2010).

Given the different roles of males and females in reproduction, reproductive traits and functions show among the highest levels of sexual dimorphism across taxa, being mostly expressed in a sex‐limited way. Whereas many studies have investigated how selection acts on reproductive traits within a sex, few have quantified the consequences of such sex‐limited selection for either the reproductive function (Fischer et al. 2009) or fitness of the other sex (Mills et al. 2012). Estimating such intersexual effects on evolutionary trajectories, however, is crucial for our understanding of how male and female fitness evolves (Cox 2014). If strong sexual dimorphism reflects the independent genetic basis of male and female reproductive function (Chippindale and Rice 2001; Postma et al. 2011; Moghadam et al. 2012; Tschirren et al. 2016), then sex‐limited selection in one sex will have little impact on the fitness of the other sex. However, if sexually dimorphic reproductive function has a shared developmental (Gilbert 2000; Smith and Sinclair 2004) and/or genetic basis (Land 1973; Simmons 2003; Fischer et al. 2009) then the intersexual consequences of sex‐limited selection on the fitness of the other sex may be substantial.

Egg production is a female‐specific reproductive trait, arising from a strong sexual dimorphism in gonadal morphology. The size of the egg represents the quantity of prenatal resource investment a female makes into each offspring and, in many taxa, has been found to have a strong impact on offspring fitness (McGinley et al. 1987; Fox and Czesak 2000; Krist 2011). Divergent artificial selection for female egg investment in a precocial bird, the Japanese quail (*Coturnix japonica*; Pick et al. 2016c), resulted in a strong divergence in female reproductive investment (seen as a strong difference in egg size, but no change in the number of eggs laid; Pick et al. 2016c), and, consequently, a pronounced difference in offspring growth and survival between the lines for high and low maternal investment (Pick et al. 2016a). Differential maternal egg investment thus directly affects female reproductive success. These artificial selection lines therefore provide an opportunity to test how directional sex‐limited selection on a key female reproductive trait affects male reproductive success. To this end, male reproductive success was assessed in both a noncompetitive mating situation, in which male–female pairs were kept in isolation (i.e., no male–male competition or female mate choice), and in a competitive mating situation under seminatural conditions that allowed for pre‐ and postcopulatory female choice and male–male competition. Furthermore, to unravel the mechanisms underlying an intersexual genetic correlation in sex‐limited reproductive function, we measured testis size and asymmetry in all males to explore the role of gonadal development in mediating intersexual fitness consequences of sex‐limited selection.

## Results and Discussion

**Figure 1:**
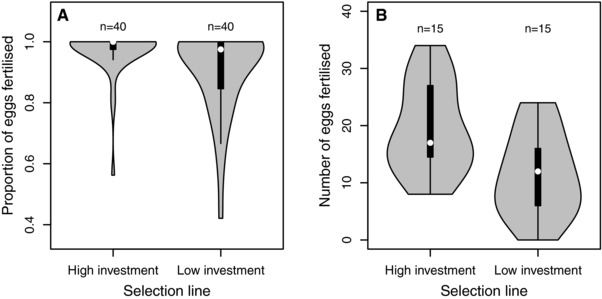
Consequences of divergent selection for female reproductive investment on (A) male fertility and (B) male reproductive success. Black boxes represent 25% and 75% quantiles, whiskers 1.5 interquartile range, and the white points the median. Kernel density plots have been added to better show the distribution of the data.

Alongside the positive consequences of selection for increased female reproductive investment on female reproductive success (measured as offspring growth and survival; Pick et al. 2016a), this sex‐limited selection also positively affected male reproductive success in both a non‐competitive (χ^2^ = 10.92, *P* = 0.001) and a competitive mating situation (*F*_1,24_ = 5.77, *P* = 0.024; Fig. 1). This finding highlights that key components of male and female fitness are positively genetically correlated. Such a positive genetic correlation between male and female fitness will amplify the evolutionary response to (sex‐specific) selection in both sexes. At the same time, a shared genetic architecture of key fitness components means that constraints acting in one sex (e.g., a trade‐off between male reproductive success and life span; Bonduriansky et al. 2008) will constrain evolution in the other (Cox 2014).

Males from lines selected for high female reproductive investment experienced higher reproductive success in both a competitive and a non‐competitive mating situation (Fig. 1). Although reproductive success in a competitive mating situation includes pre‐ and postcopulatory male–male competition and female mate choice, fertilization success in a non‐competitive environment measures only male fertility. Therefore, it is highly probable that post‐ rather than precopulatory mechanisms, and in particular sperm characteristics (e.g., ejaculate volume or sperm quality), mediate the higher reproductive success of males originating from lines selected for increased female reproductive investment. First, because even in the non‐competitive environment (no competition or choice), males originating from lines selected for high female reproductive investment fertilize more eggs, and second, because levels of sperm competition were extremely high in the competitive mating environment (multiple paternity was observed in all clutches). Interestingly, also in other taxa, the same sperm characteristics appear to influence both male fertility and success in sperm competition (Gomendio et al. 2007). Furthermore, there was no significant effect of body size on reproductive success in the competitive mating environment (*F*_1,24_ = 1.58, *P* = 0.218), which would likely reflect the ability of a male to monopolize females.

**Figure 2:**
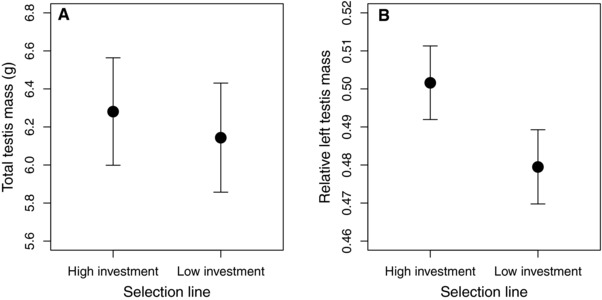
Consequences of divergent selection for female reproductive investment for male testis morphology. Differences in (A) total testis mass and (B) testis asymmetry (i.e., left testis mass as a proportion of total testis mass) between lines selected for divergent female reproductive investment. Means ± 95% CI are presented.

Male reproductive success is often linked to variation in testis size (e.g., Preston et al. 2003), as larger testes are assumed to produce more sperm (Knight 1977; Møller 1988, 1989; Calhim & Birkhead 2009). However, despite a clear difference in male reproductive success between the selection lines, there was no significant difference in total testis mass (*F*_1,104_ = 0.51, *P* = 0.478; Fig. 2A see also Fischer et al. 2009), and no significant relationship between total testis mass and fertilization success in either the competitive (*F*_1,22_ = 0.75, *P* = 0.396) or non‐competitive (χ^2^ = 0.11, *P* = 0.740) mating environment. These findings are in line with recent evidence showing that testis composition (i.e., the density of sperm‐producing tissue) is potentially as (or even more) important than their size in determining the volume and/or quality of sperm produced (Ramm and Schärer 2014; Firman et al. 2015).

Whereas there was no difference in overall testis size, males of the divergent selection lines did differ in the relative size of their left testis (*F*_1,104_ = 16.39, *P* < 0.001; Fig. 2B), often referred to as testis asymmetry (Calhim and Montgomerie 2015). This asymmetry was not simply a representation of asymmetry in the whole body, as asymmetry in the pectoral muscles did not differ between the divergent selection lines (*F*_1,76_ = 0.27, *P* = 0.602). Testis asymmetry is widespread in birds (Calhim and Montgomerie 2015), as well as other taxa (Yu 1998), but the causes of variation in testis asymmetry are not well understood (Calhim and Montgomerie 2015). As male and female gonads differentiate from the same tissue (Smith and Sinclair 2004), it has been suggested that this asymmetry (which is predominantly left‐biased across bird species; Calhim and Montgomerie 2015) is a by‐product of female birds developing reproductive organs only on their left side (Stanley and Witschi 1940) as an adaptation for flight (Zheng et al. 2013). In a previous study (Pick et al. 2016b), we demonstrated that selection for increased reproductive investment led to larger (left‐side restricted) reproductive organ size in females. In conjunction with the results presented here, this indicates the presence of a positive, intersexual genetic correlation between male and female gonadal asymmetry. The pattern of asymmetry in other taxa provides further evidence for such an intersexual genetic correlation; species in the genus *Accipiter*, for example, have a larger right testis as well as greater development of the right ovary (Stanley and Witschi 1940). Similarly, the American Alligator (*Alligator mississippiensis*), a sister taxon to birds, has larger right gonads in both sexes (Lance 1989).

**Figure 3:**
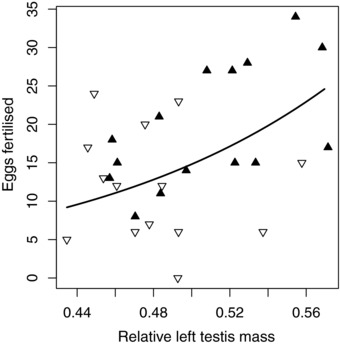
The association between testis asymmetry (i.e., left testis mass as a proportion of total testis mass) and male reproductive success, measured as the number of eggs each male fertilized in a competitive mating situation. Symbols represent the selection line (black triangles—high investment; white inverted triangles—low investment), the line represents model predictions; see Table S2b for model details.

Testis asymmetry did not only differ between the divergent selection lines, but also explained a significant amount of variation in male reproductive success both in the non‐competitive (χ^2^ = 4.76, *P* = 0.029) and the competitive (*F*_1,22_ = 7.89, *P* = 0.010; Fig. 3) mating situation. This is, to our knowledge, the first demonstration of a relationship between testis asymmetry and reproductive success in any taxon. It suggests that the two testes are functionally different, with the left being more functional than the right. Recent studies have shown that after gonadal differentiation, stem cell numbers, and gene expression profiles are higher in the left gonad in both sexes (Intarapat and Stern 2013, 2014). This difference in early development may translate into a functional difference in adulthood, resulting in a higher functionality in the left gonad in both sexes. Given that gonadal asymmetry differed between the selection lines in both sexes, selection for increased female reproductive investment has likely acted on this shared asymmetrical gonadal development. It is important to note, however, that this association between testis asymmetry is correlative, and so caution should be taken in its interpretation. Further work, linking asymmetry to testis function will allow us to fully understand the importance of this pattern.

In conclusion, we provide experimental evidence for an intersexual genetic correlation between functionally different reproductive organs, which mediates a positive genetic correlation between key fitness components in males and females. This is, to our knowledge, the first study to link such a correlated response in reproductive morphology to fitness and suggests that selection on reproductive function is acting sexually concordantly.

## Methods

For this study we used a population of Japanese quail maintained at the University of Zurich, Switzerland. We established replicated divergent selection lines for high and low maternal egg investment in this population as described in Pick et al. (2016c). In short, eggs from the 25% of females producing the largest and smallest eggs relative to their body size were incubated to create the high and low investment lines, respectively. In subsequent generations we selected the 50% of females with the most extreme egg phenotype within their respective line. We repeated this procedure twice to create two independent replicates per line (see Pick et al. 2016c for more details about the selection procedure and responses to selection). We found a strong response to selection in egg size, as well as a positive correlated response in dried egg components (i.e., in resource investment), but no response in laying rate (Pick et al. 2016c). By generation four, the lines differed in absolute egg size by 1.06 standard deviations (mean ± SDs: high investment line = 12.46 ± 0.94 g, low investment line = 11.12 ± 0.91 g). Mating between relatives were avoided throughout the selection experiment (inbreeding coefficients of individuals used in this study *f* < 0.016; Pick et al. 2016c).

We quantified variation in reproductive success of males from the third and fourth generation of these lines, in both a non‐competitive and a competitive mating situation. In the noncompetitive setting (i.e., no male–male competition or female mate choice), males from the high (*N* = 40) and low (*N* = 40) female reproductive investment lines were each mated with a female from both lines in two subsequent breeding rounds (see Supporting Information Methods for further details on mating design).

Male–female pairs were kept in individual cages. Tarsus length, a measure of body size in birds, was measured (to the nearest 0.1 mm) when the animals were brought into breeding cages. From each pair, we collected and incubated seven to 11 eggs (see Supporting Information Methods for details). All unhatched eggs were dissected and evidence of fertilization was determined visually. After breeding, all males were euthanized and their left and right testes were weighed (to nearest 0.01 g). To distinguish between testis asymmetry and whole body asymmetry, we also dissected and weighed the left and right pectoral muscles (to nearest 0.01 g). Within a replicate, all males were of the same age and had experienced the same period of reproductive activity.

Male reproductive success in a competitive setting was determined by keeping four mixed‐line groups of six to eight males and eight to 12 females (always more females than males and an equal split of males and females from each line) under seminatural conditions in large aviaries (see Supporting Information Methods for further details). This setup allowed for pre‐ and postcopulatory female mate choice and male–male competition. We measured tarsus length and took a small blood sample from all animals when they entered the aviary. This blood sample was stored in 96% ethanol for parentage assignment. Over a period of 14–16 days, all eggs were collected daily from the aviaries and incubated for four to six days. Embryonic tissue was collected from the developing eggs and stored in 96% ethanol. DNA was extracted and parentage was assigned using microsatellite markers (see Supporting Information Methods for further details). Males were subsequently euthanized, and their left and right testes were weighed (to nearest 0.01 g). All procedures were conducted under licenses provided by the Veterinary Office of the Canton of Zurich, Switzerland (permit numbers 195/2010; 14/2014; 156).

### STATISTICAL ANALYSES

We tested for a difference in male fertilization success in the non‐competitive mating situation using a generalized linear mixed model with a binomial error distribution and a logit link function, in which each egg was scored as either fertilized or unfertilized. Selection line of the male and female, and selection line replicate were included as fixed factors, egg number (in laying sequence) and body size (tarsus length) as a covariate, and male and female identity as random effects. Tarsus length was *z*‐transformed before analysis, to aid in model convergence. For this analysis we included only males that bred twice with selection line females (*N* = 77 males; 154 pairings; 1449 eggs; see Supporting Information Methods). We further tested for an effect of total testis mass (summed mass of left and right testes) and testis asymmetry (left testis as proportion of total testis mass) on fertilization success using the same models as outlined above, but including testis mass and asymmetry instead of selection line, and using data from all pairings (*N* = 80 males; 160 pairings; 1503 eggs).

We tested for a difference in the number of eggs fertilized by a male in the competitive mating situation using generalized linear models with a Poisson error distribution and log link function, using quasilikelihood to correct for overdispersion. These models included male selection line and aviary ID as fixed factors, and body size (tarsus length) as a covariate (*N* = 30 males). We further tested for an effect of total testis mass and testis asymmetry on the number of eggs fertilized using the same models, but including total testis mass and asymmetry instead of selection line (*N* = 29 males; the testes of one male were not measured).

Finally, we tested for a difference between the selection lines in total testis mass and testis asymmetry using linear models that included selection line, generation and line replicate as factors, and tarsus length as a covariate (*N* = 109 males). To distinguish between testis asymmetry and whole‐body asymmetry, we ran the same model with pectoral asymmetry, excluding generation (pectoral muscle data were only available from males in the noncompetitive mating situation; *N* = 80 males).

Significance was determined in (generalized) linear models using *F* statistics and in mixed models by comparing nested models using likelihood‐ratio tests, for which df = 1. Analyses were performed in R (3.3.0; R Core Team 2016). Mixed models were performed using lme4 (1.1‐12; Bates et al. 2015). Detailed results of all models are provided in the Supporting Information Results.

Associate Editor: A. Gardner

Handling Editor: J. Slate

## Supporting information


**Table S1**. Effects of (a) male selection line and (b) testis morphology on male fertilization success in a noncompetitive mating situation.
**Table S2**. Effects of (a) male selection line and (b) testis morphology on male reproductive success in a competitive mating situation.
**Table S3**. Effects of male selection line on (a) total testis mass, (b) testis asymmetry, and (c) pectoral muscle asymmetry.

## References

[evl321-bib-0001] Bates, D. , M. Mächler , B. Bolker , and S. Walker . 2015 Fitting linear mixed‐effects models using {lme4}. J. Stat. Softw. 67:1–48.

[evl321-bib-0002] Bonduriansky, R. , and S. F. Chenoweth . 2009 Intralocus sexual conflict. Trends Ecol. Evol. 24:280–288. 19307043 10.1016/j.tree.2008.12.005

[evl321-bib-0003] Bonduriansky, R. , A. Maklakov , F. Zajitschek , and R. Brooks . 2008 Sexual selection, sexual conflict and the evolution of ageing and lifespan. Funct. Ecol. 22:443–453.

[evl321-bib-0004] Bonduriansky, R. , and L. Rowe . 2005 Intralocus sexual conflict and the genetic architecture of sexually dimorphic traits in *Prochyliza xanthostoma* (Diptera: Piophilidae). Evolution 59:1965–1975. 16261734

[evl321-bib-0005] Calhim, S. , and T. R. Birkhead . 2009 Intraspecific variation in testis asymmetry in birds: evidence for naturally occurring compensation. Proc. R. Soc. Lond. B 276:2279–2284. 10.1098/rspb.2009.0134 PMC2677609 19324740

[evl321-bib-0006] Calhim, S. , and R. Montgomerie . 2015 Testis asymmetry in birds: the influences of sexual and natural selection. J. Avian Biol. 46:175–185.

[evl321-bib-0007] Chippindale, A. K. , J. R. Gibson , and W. R. Rice . 2001 Negative genetic correlation for adult fitness between sexes reveals ontogenetic conflict in *Drosophila* . Proc. Natl. Acad. Sci. USA 98:1671–1675. 11172009 10.1073/pnas.041378098 PMC29315

[evl321-bib-0008] Chippindale, A. K. , and W. R. Rice . 2001 Y chromosome polymorphism is a strong determinant of male fitness in *Drosophila melanogaster* . Proc. Natl. Acad. Sci. USA 98:5677–5682. 11320221 10.1073/pnas.101456898 PMC33272

[evl321-bib-0009] Cox, R. M. 2014 Integrating costs of reproduction between the sexes Pp. 153–168 *in* Martin L. B., Ghalambor C. K., and Woods H. A., eds. Integrative organismal biology. John Wiley & Sons, Inc, Hoboken, NJ.

[evl321-bib-0010] Cox, R. M. , and R. Calsbeek . 2009 Sexually antagonistic selection, sexual dimorphism, and the resolution of intralocus sexual conflict. Am. Nat. 173:176–187. 19138156 10.1086/595841

[evl321-bib-0011] Cox, R. M. , C. L. Cox , J. W. McGlothlin , D. C. Card , A. L. Andrew , and T. A. Castoe . 2017 Hormonally mediated increases in sex‐biased gene expression accompany the breakdown of between‐sex genetic correlations in a sexually dimorphic lizard. Am. Nat. 189:315–332. 28221827 10.1086/690105

[evl321-bib-0012] Coyne, J. A. , E. H. Kay , and S. Pruett‐Jones . 2008 The genetic basis of sexual dimorphism in birds. Evolution 62:214–219. 18005159 10.1111/j.1558-5646.2007.00254.x

[evl321-bib-0013] Fedorka, K. M. , and T. A. Mousseau . 2004 Female mating bias results in conflicting sex‐specific offspring fitness. Nature 429:65–67. 15129280 10.1038/nature02492

[evl321-bib-0014] Firman, R. C. , F. Garcia‐Gonzalez , E. Thyer , S. Wheeler , Z. Yamin , M. Yuan , and L. W. Simmons . 2015 Evolutionary change in testes tissue composition among experimental populations of house mice. Evolution 69:848–855. 25600637 10.1111/evo.12603

[evl321-bib-0015] Fischer, K. , K. Zimmer , and N. Wedell . 2009 Correlated responses to selection on female egg size in male reproductive traits in a butterfly. Evol. Ecol. 23:389–402.

[evl321-bib-0016] Foerster, K. , T. Coulson , B. C. Sheldon , J. M. Pemberton , T. H. Clutton‐Brock , and L. E. B. Kruuk . 2007 Sexually antagonistic genetic variation for fitness in red deer. Nature 447:1107–1110. 17597758 10.1038/nature05912

[evl321-bib-0017] Fox, C. W. , and M. E. Czesak . 2000 Evolutionary ecology of progeny size in arthropods. Annu. Rev. Entomol. 45:341–369. 10761581 10.1146/annurev.ento.45.1.341

[evl321-bib-0018] Gilbert, S. F. 2000 Chromosomal sex determination in mammals Developmental biology. 6th ed. Sinauer Associates, Sunderland, MA.

[evl321-bib-0019] Gomendio, M. , A. F. Malo , J. Garde , and E. R. S. Roldan . 2007 Sperm traits and male fertility in natural populations. Reproduction 134:19–29. 17641085 10.1530/REP-07-0143

[evl321-bib-0020] Harano, T. , K. Okada , S. Nakayama , T. Miyatake , and D. J. Hosken . 2010 Intralocus sexual conflict unresolved by sex‐limited trait expression. Curr. Biol. 20:2036–2039. 21055943 10.1016/j.cub.2010.10.023

[evl321-bib-0021] Intarapat, S. , and C. D. Stern . 2013 Sexually dimorphic and sex‐independent left‐right asymmetries in chicken embryonic gonads. PLoS One 8:1–8. 10.1371/journal.pone.0069893 PMC3716703 23894556

[evl321-bib-0022] Intarapat, S. , and C. D. Stern 2014 Left‐right asymmetry in chicken embryonic gonads. J. Poult. Sci. 51:352–358.

[evl321-bib-0023] Knight, T. W. 1977 Methods for the indirect estimation of testes weight and sperm numbers in Merino and Romney rams. N. Z. J. Agric. Res. 20:291–296.

[evl321-bib-0024] Krist, M. 2011 Egg size and offspring quality: a meta‐analysis in birds. Biol. Rev. Camb. Philos. Soc. 86:692–716. 21070586 10.1111/j.1469-185X.2010.00166.x

[evl321-bib-0025] Lance, V. 1989 Reproductive cycle of the American alligator. Am. Zool. 29:999–1018.

[evl321-bib-0026] Land, R. B. 1973 The expression of female sex‐limited characters in the male. Nature 241:208–209. 4700891 10.1038/241208a0

[evl321-bib-0027] Lande, R. 1979 Sexual dimorphism, sexual selection, and adaptation in polygenic characters. Evolution 34:292–305. 10.1111/j.1558-5646.1980.tb04817.x 28563426

[evl321-bib-0028] McGinley, M. , D. Temme , and M. Geber . 1987 Parental investment in offspring in variable environments: theoretical and empirical considerations. Am. Nat. 130:370–398.

[evl321-bib-0029] Mills, S. C. , E. Koskela , and T. Mappes . 2012 Intralocus sexual conflict for fitness: sexually antagonistic alleles for testosterone. Proc. R. Soc. Lond. B, 279, 1889–1895. 10.1098/rspb.2011.2340 PMC3311893 22171083

[evl321-bib-0030] Moghadam, H. , M. Pointer , A. Wright , S. Berlin , and J. Mank . 2012 W chromosome expression responds to female‐specific selection. Proc. Natl. Acad. Sci. USA 109:8207–8211. 22570496 10.1073/pnas.1202721109 PMC3361381

[evl321-bib-0031] Møller, A. P. 1988 Testes size, ejaculate quality and sperm competition in birds. Biol. J. Linn. Soc. 33:273–283.

[evl321-bib-0032] Møller, A. P. 1989 Ejaculate quality, testes size and sperm production in mammals. Funct. Ecol. 3:91–96.

[evl321-bib-0033] Pick, J. L. , C. Ebneter , P. Hutter , and B. Tschirren . 2016a Disentangling genetic and prenatal maternal effects on offspring size and survival. Am. Nat. 188:628–639. 27860503 10.1086/688918

[evl321-bib-0034] Pick, J. L. , P. Hutter , C. Ebneter , A. K. Ziegler , M. Giordano , and B. Tschirren . 2016b Artificial selection reveals the energetic expense of producing larger eggs. Front. Zool. 13:38. 27559356 10.1186/s12983-016-0172-y PMC4995767

[evl321-bib-0035] Pick, J. L. , P. Hutter , and B. Tschirren . 2016c In search of genetic constraints limiting the evolution of egg size: direct and correlated responses to artificial selection on a prenatal maternal effector. Heredity 116:542–549. 26956564 10.1038/hdy.2016.16 PMC4868267

[evl321-bib-0049] Pick, J. L. , P. Hutter , and B. Tschirren . 2017 Divergent artificial selection for female reproductive investment has a sexually concordant effect on male reproductive success. Dryad Digital Repository. 10.5061/dryad.164m0. PMC6121851 30283651

[evl321-bib-0036] Poissant, J. , A. J. Wilson , and D. W. Coltman 2010 Sex‐specific genetic variance and the evolution of sexual dimorphism: a systematic review of cross‐sex genetic correlations. Evolution 64:97–107. 19659596 10.1111/j.1558-5646.2009.00793.x

[evl321-bib-0037] Postma, E. , N. Spyrou , L. A. Rollins , and R. C. Brooks . 2011 Sex‐dependent selection differentially shapes genetic variation on and off the guppy Y chromosome. Evolution 65:2145–2156. 21790565 10.1111/j.1558-5646.2011.01314.x

[evl321-bib-0038] Preston, B. T. , I. R. Stevenson , J. M. Pemberton , D. W. Coltman , and K. Wilson . 2003 Overt and covert competition in a promiscuous mammal: the importance of weaponry and testes size to male reproductive success. Proc. R. Soc. Lond. B 270:633–640. 10.1098/rspb.2002.2268 PMC1691283 12769464

[evl321-bib-0039] R Core Team . 2016 R: a language and environment for statistical computing. R Foundation for Statistical Computing, Vienna, Austria.

[evl321-bib-0040] Ramm, S. A. , and L. Schärer . 2014 The evolutionary ecology of testicular function: size isn't everything. Biol. Rev. Camb. Philos. Soc. 89:874–888. 24495304 10.1111/brv.12084

[evl321-bib-0041] Simmons, L. W. 2003 The evolution of polyandry: patterns of genotypic variation in female mating frequency, male fertilization success and a test of the sexy‐sperm hypothesis. J. Evol. Biol. 16:624–634. 14632226 10.1046/j.1420-9101.2003.00572.x

[evl321-bib-0042] Smith, C. A. , and A. H. Sinclair . 2004 Sex determination: insights from the chicken. BioEssays 26:120–132. 14745830 10.1002/bies.10400

[evl321-bib-0043] Stanley, A. , and E. Witschi . 1940 Germ cell migration in relation to asymmetry in the sex glands of hawks. Anat. Rec. 76:329–342.

[evl321-bib-0044] Tschirren, B. , A. Ziegler , J. Pick , M. Okuliarová , M. Zeman , and M. Giraudeau . 2016 Matrilineal inheritance of a key mediator of prenatal maternal effects. Proc. R. Soc. Lond. B 238:20161676. 10.1098/rspb.2016.1676 PMC5031669 27629040

[evl321-bib-0045] Whitlock, M. C. , and A. F. Agrawal . 2009 Purging the genome with sexual selection: reducing mutation load through selection on males. Evolution 63:569–582. 19154364 10.1111/j.1558-5646.2008.00558.x

[evl321-bib-0046] Yu, Z. H. 1998 Asymmetrical testicular weights in mammals, birds, reptiles and amphibia. Int. J. Androl. 21:53–55. 9639153 10.1046/j.1365-2605.1998.00088.x

[evl321-bib-0047] Zheng, X. , J. O'Connor , F. Huchzermeyer , X. Wang , Y. Wang , M. Wang , and Z. Zhou . 2013 Preservation of ovarian follicles reveals early evolution of avian reproductive behaviour. Nature 495:507–511. 23503663 10.1038/nature11985

