# Supplementary material for: Divergent artificial selection for female reproductive investment has a sexually concordant effect on male reproductive success
Source: Evol Lett. 2017 Aug 23;1(4):222–8. doi: 10.1002/evl3.21 (PMC6121851; doi:10.1002/evl3.21)
Supplement: Supplementary file 1 — Table S1. Effects of (a) male selection line and (b) testis morphology on male fertilization success in a noncompetitive mating situation. Table S2. Effects of (a) male selection line and (b) testis morphology on male reproductive success in a competitive mating situation. Table S3. Effects of male selection line on (a) total testis mass, (b) testis asymmetry, and (c) pectoral muscle asymmetry. [file EVL3-1-222-s001.pdf]

Divergent artificial selection for female reproductive investment has a sexually concordant effect on male reproductive success

Pick, J.L., Hutter, P., & Tschirren, B

Supplementary Materials

Supplementary Methods

Non-competitive mating design

80 males (20 from each line replicate) from the fourth generation of selection were each mated with two females, one from each selection line, in different breeding rounds (N = 80 females; 20 from each line replicate). The order of matings (same vs. different line) was randomized. To control matings, male-female pairs were brought into breeding cages (122 x 50 x 50cm) inside our breeding facility for 25 days, with one male-female pair per cage. As a measure of body size, tarsus length was measured (to the nearest 0.1mm) when the birds were brought into breeding cages. Our facility is kept at approximately 20°C, on a 16h:8h light:dark cycle, which is sufficient to keep the birds in breeding condition (6). Quails had access to *ad libitum* food, water, grit, and a source of calcium. Cages also contained a house and a raised sand bath, and the bottom of the cages was filled with sawdust. Between breeding rounds, individuals spent six to eight weeks in outdoor aviaries (5.5 x 7m); females in a single sex aviary, males in a mixed sex aviary with non-experimental females. This ensured that sperm from previous matings was depleted (females can store sperm for up to 11 days; 7, PH unpublished data). Five females (one high investment, four low investment), but no males, died between breeding rounds. These females were replaced between the two breeding rounds, to ensure each male bred with two different females. One female was replaced by another female from the same selection line, the others by a female from an unselected base population. Eggs were collected daily from each cage. From each pair seven to 11 eggs were stored at 12°C (for a maximum of four days) before being artificially incubated (Favorit, HEKA Brutgeräte, Rietberg, Germany) at 37.8°C and 55% humidity, in two sets of up to six eggs. After breeding for the second time, all males were euthanised with a CO_2 and argon mix (CO_2 - 31%, Ar - 67%, O_2 - 2%) between 08:00 and 09:00.

Competitive mating design

We used four mixed line groups of six or eight males and eight or 12 females (always more females than males and an equal split of males and females from each line; in total 44 females and 30 males). Quail were taken from the third and fourth generation of the selection experiment, but each group contained animals from only one generation and one selection line replicate. The groups were kept in indoor aviaries (232x180cm), which contained wooden shelters (24x35x30 cm). All animals had access to *ad libitum* food and water and to a sand bath. They could move freely within the aviary and interact with all other individuals present. When the animals entered the aviary, we measured their tarsus length and took a small blood sample, which was stored in 96% ethanol at -20°C for parentage assignment. The females were released into the aviary eight to 12 days before the experiment started to habituate them to the new environment. After the introduction of males into the aviary, all eggs were collected each morning for 14 to 16 days. All eggs were incubated for four-six days, at which point any embryonic tissue was removed and stored in 96% ethanol at -20°C. Eggs collected on the two days immediately after introduction of the males into the aviary had extremely high level of infertility (100% and 59% respectively), as the fertile period for these eggs would have been missed in the majority of females. We therefore excluded these eggs from the analysis.

Parentage Assignment

DNA from blood and tissue samples was extracted using standard protocols (5). We used nine microsatellite makers, split in two panels (Panel 1: GUJ0024, GUJ0029, GUJ0068, GUJ0085; Panel 2: 01_089677546, 02_001318108, 13_011188518, 14_007438907, GUJ0023 (2–4)), to assign parentage. Amplifications were carried out in a reaction volume of 10 ul, including Multiplex PCR Master Mix (Qiagen AG, Basel,

Switzerland), fluorescent-labeled forward primers and non-labeled reverse primers on a GeneAmp PCR System 9700 thermocyler (Applied Biosystems, Rotkreuz, Switzerland). The PCR protocol started with an initial denaturation step at 95°C for 15 min, followed by 30 cycles of 30s at 94°C, 90s at 54°C for Panel 1 and 55°C for Panel 2, 60s at 72°C, followed by a final extension step at 60°C for 30min. Fragments were separated on a ABI Prism 3730 capillary sequencer (Applied Biosystems, Rotkreuz, Switzerland) and analysed in GeneMapper v. 3.7 (Applied Biosystems, Rotkreuz, Switzerland).

We calculated allele frequencies and exclusion probabilities for each group separately using Cervus v. 3.0.3 (1). The mean exclusion probability for the first parent was 0.830 (range: 0.812-0.870) and for the second parent 0.970 (range: 0.965-0.980), with a mean number of alleles of 4.4 (range 2-7). For the parentage assignment we used trio logarithm of the odds (LOD) scores and confirmed the assignment by exclusion. In total we collected 505 eggs, and successfully extracted DNA from and assigned parents to 464 developing embryos.

Supplementary Results

Table S1: Effects of (a) male selection line and (b) testis morphology on male fertilisation success in a non-competitive mating situation. The factor level of comparison is indicated in brackets.

a)

Predictor	Estimate	SE	χ^2	p
Intercept	5.10	1.84	-	-
Male selection line (Low)	-2.66	0.84	10.92	0.001
Female selection line (Low)	0.51	0.78	0.43	0.511
Replicate (2)	0.84	0.95	0.76	0.383
Egg number	0.16	0.05	9.98	0.002
Tarsus length	-0.61	0.45	1.90	0.168

b)

Predictor	Estimate	SE	χ^2	p
Intercept	-9.46	6.08	-	-
Testis size	0.15	0.45	0.11	0.740
Testis asymmetry	24.43	11.28	4.76	0.029
Replicate (2)	1.40	1.00	1.90	0.168
Egg number	0.16	0.05	10.09	0.001
Tarsus length	0.11	0.41	0.07	0.796

Table S2: Effects of (a) male selection line and (b) testis morphology on male reproductive success in a competitive mating situation. The factor level of comparison is indicated in brackets.

a)

Predictor	Estimate	SE	*F*	DF	*p*
Intercept	-1.58	3.44	-	-	-
Male selection line (Low)	-0.49	0.20	5.768	1, 24	0.024
Aviary (2)	0.00	0.28	0.046	3, 24	0.986
Aviary (3)	-0.01	0.30	-	-	-
Aviary (4)	0.08	0.27	-	-	-
Tarsus length	0.11	0.09	1.597	1, 24	0.218

b)

Predictor	Estimate	SE	*F*	DF	*p*
Intercept	-7.43	3.55	-	-	-
Testis size	0.10	0.11	0.749	1, 22	0.396
Testis asymmetry	7.52	2.56	7.892	1, 22	0.010
Aviary (2)	0.02	0.28	0.914	3, 22	0.450
Aviary (3)	-0.32	0.33	-	-	-
Aviary (4)	0.19	0.29	-	-	-
Tarsus length	0.15	0.08	3.041	1, 22	0.095

Table S3: Effects of male selection line on (a) total testis mass, (b) testis asymmetry and (c) pectoral muscle asymmetry. The factor level of comparison is indicated in brackets.

a)

Predictor	Estimate	SE	F	DF	p
Intercept	5.46	3.11	-	-	-
Male selection line (Low)	-0.14	0.20	0.508	1, 104	0.478
Replicate (2)	-0.19	0.20	0.879	1, 104	0.351
Generation (4)	1.38	0.28	23.602	1, 104	0.000
Tarsus length	-0.01	0.08	0.008	1, 104	0.928

b)

Predictor	Estimate	SE	F	DF	p
Intercept	0.82	0.11	-	-	-
Male selection line (Low)	-0.03	0.01	16.391	1, 104	0.000
Replicate (2)	-0.02	0.01	4.417	1, 104	0.038
Generation (4)	0.01	0.01	0.400	1, 104	0.528
Tarsus length	-0.01	0.00	8.221	1, 104	0.005

c)

Predictor	Estimate	SE	F	DF	p
Intercept	0.54	0.03	-	-	-
Male selection line (Low)	0.00	0.00	0.274	1, 76	0.602
Replicate (2)	0.00	0.00	0.066	1, 76	0.798
Tarsus length	0.00	0.00	1.349	1, 76	0.249

References

[1] Kalinowski, S.T., Taper, M.L. & Marshall, T.C. (2007). Revising how the computer program CERVUS accommodates genotyping error increases success in paternity assignment. *Mol. Ecol.* 16:1099–1106.

[2] Kawahara-Miki, R., Sano, S., Nunome, M., Shimmura, T., Kuwayama, T., Takahashi, S. *et al.* (2013). Next-generation sequencing reveals genomic features in the Japanese quail. *Genomics.* 101:345–53.

[3] Kayang, B.B., Inoue-Murayama, M., Nomura, A., Kimura, K., Takahashi, H., Mizutani, M. *et al.* (2000). Fifty microsatellite markers for Japanese quail. *J. Hered.* 91:502–505.

[4] Kayang, B.B., Noue-Murayama, M., Hoshi, T., Matsuo, K., Takahashi, H., Minezawa, M. *et al.* (2002). Microsatellite loci in Japanese quail and cross-species amplification in chicken and guinea fowl. *Genet. Sel. Evol.* 34:233–253.

[5] Laird, P.W., Zijderveld, A., Linders, K., Rudnicki, M.a., Jaenisch, R. & Berns, A. (1991). Simplified mammalian DNA isolation procedure. *Nucleic Acids Res.* 19:4293.

[6] Sachs, B.D. (1969). Photoperiodic control of reproductive behavior and physiology of the male Japanese quail (*Coturnix coturnix japonica*). *Horm. Behav.* 24:7–24.

[7] Sittmann, K. & Abplanalp, H. (1965). Duration and recovery of fertility in Japanese quail (*Coturnix coturnix japonica*). *Br. Poult. Sci.* 6:245–250.
